# Eliminating Healthcare-Associated Infections in Iran: A Qualitative Study to Explore Stakeholders’ Views

**DOI:** 10.15171/ijhpm.2017.34

**Published:** 2017-04-15

**Authors:** Atefeh Esfandiari, Hedayat Salari, Arash Rashidian, Hossein Masoumi Asl, Abbas Rahimi Foroushani, Ali Akbari Sari

**Affiliations:** ^1^ Bushehr University of Medical Sciences, Bushehr, Iran.; ^2^ Department of Health Management and Economics, School of Public Health, Tehran University of Medical Sciences, Tehran, Iran.; ^3^ Center for Communicable Diseases Control, Ministry of Health and Medical Education, Tehran, Iran.; ^4^ Research Center of Pediatric Infectious Diseases, Rasoul-e-Akram Hospital, Iran University of Medical Sciences, Tehran, Iran.; ^5^ Department of Epidemiology and Biostatistics, School of Public Health, Tehran University of Medical Sciences, Tehran, Iran.

**Keywords:** Healthcare-Associated Infection (HAI), Elimination, Stakeholders’ Views, Policy Implications, Qualitative Study, Iran

## Abstract

**Background:** Although preventable, healthcare-associated infections (HAIs) continue to pose huge health and economic burdens on countries worldwide. Some studies have indicated the numerous causes of HAIs, but only a tiny literature exists on the multifaceted measures that can be used to address the problem. This paper presents stakeholders’ opinions on measures for controlling HAIs in Iran.

**Methods:** We used the qualitative research method in studying the phenomenon. Through a purposive sampling approach, we conducted 24 face-to-face interviews using a semi-structured interview guide. Participants were mainly key informants, including policy-makers, health professionals, and technical officers across the national and subnational levels, including the Ministry of Health (MoH), medical universities, and hospitals in Iran. We performed thematic framework analysis using the software MAXQDA10.

**Results:** Four main interdisciplinary themes emerged from our study of measures of controlling HAIs: strengthening governance and stewardship; strengthening human resources policies; appropriate prescription and usage of antibiotics; and environmental sanitation and personal hygiene.

**Conclusion:** According to our findings, elimination of HAIs demands multifactorial interventions. While the ultimate recommendation of policy-makers is to have HAIs among the priorities of the national agenda, financial commitment and the creation of an enabling work environment in which both patients and healthcare workers can practice personal hygiene could lead to a significant reduction in HAIs in Iran.

## Background

Healthcare-associated infections (HAIs) remain a major threat to public health and the economy worldwide. In the United States, for example, 1 out of every 20 hospitalized patients acquires an HAI.^1^ Recent data indicate that approximately 722 000 HAIs occurred in US acute care hospitals in the year 2011, leading to almost 75 000 deaths of hospitalized patients. Evidences show that more than half of all HAIs occur outside intensive care units (ICUs).^2^ Furthermore, hospital-acquired infections cost the US economy about US$33 billion annually.^1^ In the EU, it is estimated that over 4.5 million people are affected by HAIs yearly, leading to approximately 37 000 deaths and 16 million extra days in hospitals.^3^ The global economic burden of HAIs based on a systematic review conducted over 1986–2013 on five HAIs, namely surgical site infection (SSI), central line associated bloodstream infection (CLABSI), catheter-associated urinary tract infection (CAUTI), ventilator-associated pneumonia (VAP) and Clostridium difficile infection (CDI), was found to be US$9.8 billion.^4^

The economy of Iran is also affected by HAIs. The incidence of HAIs is nearly 18%, with the economic burden within one specific ward found to be US$150 000.^5^ The overall death rate from HAIs, according to a 5-year report (2007-2010), was found to be 14.8%,^6^ with the highest rate of HAIs among the reported cases (28.9%) related to urinary tract infections (UTIs). Most HAIs were detected in ICUs and surgery wards, with rates of 26.7% and 12.8% respectively in Iran.^7^

The increasing threat posed by HAIs has resulted in multiple control mechanisms worldwide. As an example, in the year 2005 the World Health Organization (WHO) initiated the Global Patient Safety Challenge project, with the slogan “clean care is safer care.”^8^ HAIs continue to pose significant health and economic consequences to countries worldwide, including Iran. Studies have indicated numerous causes of HAIs.^3,9^ However, only a tiny literature exists on the multifaceted interventions that could be used to address the problem in Iran. This paper aimed to explore stakeholders’ views on measures of controlling HAIs in Iran.

## Methods

A qualitative study design was used to explore the phenomenon. We employed the purposive sampling technique to identify study participants until reaching data saturation. The experts were drawn from the Ministry of Health (MoH) (10), universities (7) and hospitals (7) across three levels – macro, meso and micro – and were all involved in the prevention and control of HAIs in Iran. The interviewees were mainly key informants and representatives of the National Committee of Hospital Infection in Iran, including national policy-makers, healthcare professionals and health technical officers. Characteristics of the interviewees are indicated in Table 1.

**Table 1:** The Characteristics of the Interviewees

	**Organization**	**Level**	**Job Posting**	**Experience**
1	MoHME-Health Deputy	Macro	High level manager in Center for Communicable Diseases Control	25
2	MoHME-Health Deputy	Macro	High level manager in Center for Communicable Diseases Control	30
3	MoHME-Health Deputy	Macro	Technical officer in HAIs prevention and control	24
4	MoHME-Health Deputy	Macro	High level manager	13
5	MoHME-Deputy of Curative Affaires	Macro	Technical officer in accreditation office	10
6	MoHME-Deputy of Curative Affaires	Macro	High level manager in reference laboratory	19
7	MoHME-Deputy of Curative Affaires	Macro	High level manager in reference laboratory	15
8	MoHME-Deputy of Curative Affaires	Macro	Technical officer in charge of patient safety	20
9	MoHME-Deputy of Curative Affaires	Macro	High level manager	18
10	MoHME-Food and Drug Organization	Macro	High level manager	10
11	Medical Universities	Meso	Head of diseases control department in health deputy	12
12	Medical Universities	Meso	Technical officer in deputy of medical affaires	8
13	Medical Universities	Meso	Head of diseases control department in health deputy	14
14	Medical Universities	Meso	Head of diseases control department in health deputy	17
15	Medical Universities	Meso	Head of diseases control department in health deputy	12
16	Medical Universities	Meso	Technical officer in deputy of medical affaires	26
17	Medical Universities	Meso	Health deputy director	20
18	Hospitals	Micro	Head of infection control committee	17
19	Hospitals	Micro	Head of infectious diseases ward	25
20	Hospitals	Micro	Infection control supervisor	20
21	Hospitals	Micro	Infection control supervisor	16
22	Hospitals	Micro	Infection control supervisor	8
23	Hospitals	Micro	Nurse of ICU	16
24	Hospitals	Micro	Nurse of CCU	6

Abbreviations: MoHME, Ministry of Health and Medical Education; HIAs, Healthcare-associated infections; ICU, intensive care uni; CCU, coronary care unit.

We used a semi-structured interview guide for data collection. To develop the interview guide, we first conducted a literature review ourselves and then two face-to-face in-depth interviews to obtain expert information to complete the information from the literature. After that, 2 pilot interviews were first conducted to check the validity of our data collection instruments. In the first section in the instrument we asked some demographic questions about the age, gender, experience, organization and job role of the participants. The second part included some questions about the financial, environmental, cultural, institutional, legal, educational, pharmaceutical and personnel solutions and policies for eliminating HAIs. We also included one general question aimed at obtaining descriptive answers of any other strategy they might think of for preventing and controlling HAIs. We continued conducting interviews until saturation was achieved with the analysis and comparison of the interview content. In the end, a total of 24 face-to-face in-depth interviews were conducted from June 2014 to April 2015. Each interview lasted between 45 and 120 minutes. Most of the interviews were tape-recorded but this depended on the approval of the interviewees. Notes were taken if participants (2) did not allow the interviewers to record their voice. The recorded interviews were transcribed.

Data were coded using MAXQDA10 software. We used the thematic framework approach for data analysis. The approach consists of five steps: “familiarization,” “identification of thematic framework,” “indexing,” “charting,” and “mapping and interpretation.”^10-12^ The WHO health system framework^13,14^ served as a guide for interpreting the data. Two of the authors (A.E and H.S) immersed themselves in the data by listening to the audiotapes and reading the transcripts repeatedly. This enabled us to gain a holistic view of the issues surrounding prevention and control of HAIs. Data associated with the codes and relevant to the research objectives were compared and discussed. We then arranged the pieces of indexed data in charts - made up of the main themes and their sub-themes. Finally, core themes were selected and analyzed.

We also ensured the credibility, transferability, dependability and confirmability of our findings. Prolonged engagement (the research process lasted about 11 months) and member checks (some parts of the transcribed interviews were revised by the interviewees) were adopted to enhance the credibility of our findings. The purposive sampling technique and detailed descriptions enabled us to avoid vague statements, thereby enhancing the transferability of the data. Moreover, we cross-checked the data we gathered, performed thorough documentation and analysis of results, and also employed external reviewers to ensure the dependability and confirmability of our findings. The external reviewer was a final-year PhD candidate who had worked on health systems and medical errors and was very skilled in qualitative methods. He reviewed all the interviews and coding, checked the relationships between the research questions and the results, and looked for consistency throughout the whole project.

## Results

In this section, we describe the stakeholders’ views on measures for controlling HAIs in Iran. Four main themes were identified here: strengthening governance and stewardship; strengthening human resources policies; appropriate prescription and usage of antibiotics; and environmental sanitation and personal hygiene (Table 2).

**Table 2:** Measures for Eliminating HAIs in Iran

**Themes**	**Subthemes**	**Codes**
Theme 1: Strengthening governance and stewardship	Enhancing governance and factors related to health system	- Placing prevention and control of HAIs as a national health agenda - Structural reforms - Giving authority to infection control committees - Increasing financial and material resources - Information and reporting system strengthening - Rewards and punishments - Training/education of health care personnel
	Enhancing stewardship	- Appointing a unique steward - Improving Interdisciplinary cooperation - Influence on health care professionals behavior - Developing National guidelines and standards to control of HAIs
Theme 2: Strengthening human resources policies	Increasing Human resources	- Increasing the number of skilled nurse staffing - Increasing infectious disease physician as consultants - Increase the number of microbiologist for laboratory diagnostic services - Adequate trained housekeeping staff
	Training/education of health care personnel and People	- Education (of various forms; face to face provision of educational pamphlets and posters on infection control) - Active engagement with the media - Suggested a “Hand Hygiene Day” to raise public awareness
	Giving rewards and punishments	- Giving incentive to adhere to standards - Being punished for failure to act in accordance with principles
Theme 3: Appropriate prescription and usage of antibiotics	Antimicrobial management Programs	- Establishing an Antibiotics Stewardship Program - Developing evidence-based guidelines for prescription of antibiotic - Determining antibiotics using patterns
	Restriction of antimicrobial prescription	- Consultation with infectious diseases specialist in hospitals - Not allowing to prescribe all the antibiotics by all the physicians
Theme 4: Environmental sanitation and personal hygiene	Environmental sanitation	- Water quality - Proper ventilation for specialized care - Appropriate use of disinfectants, waste management
	Personal hygiene	- Hand Hygiene - Compliance of standards precuations

Abbreviation: HIAs, Healthcare-associated infections.

### 1. Strengthening Governance and Stewardship

#### 1-1. Enhancing Governance and Factors Related to the Health System

There are several factors related to the health system and governance that affect the prevention and control of HAIs. Making the prevention and control of HAIs part of the national health agenda, structural reforms, giving authority to infection control committees, increasing financial and material resources, and strengthening the information and reporting system are some of the options suggested by the key informants.

### Making the Prevention and Control of Healthcare-Associated Infections Part of the National Health Agenda

According to our study participants, although HAIs have huge health and economic consequences for Iran, the issue has not been made part of the national agenda. They suggested that any further reform within the health system should prioritize HAIs and their control due to their devastating and increasing consequences:

*“Now you see that there are some more important health policies included in the national agenda. Practically, although some effective strategies are being implemented in the healthcare setting for infection control, there is not enough attention paid at the national level to eliminating HAIs. Policy-makers must pay considerable attention to infection control in the country, especially in the new health plan, which is the Health Sector Evolution Plan, which started in May 2014”* (Health professional; 18).

### Structural Reforms

Reforms proposed by the policy-makers included the creation of an office to control infections instead of the infection control committee, the provision of a job description for infection control doctors and nurses, and the assignment of a national HAIs coordinator:

*“There is a need to establish an organization or entity for HAI control in hospitals, and standard processes and mechanisms should be defined for these activities in h*ospitals*. Instead of setting up a committee responsible for the control of infections, we should establish an office of infection control. With that, we could have personnel dedicated to the effective control of infections”* (Policy-maker; 1).

*“In some healthcare settings in the world, infection control physician and nurse job roles are defined, but in our country such jobs do not exist. Doctors and nurses should also be assigned some tasks that clearly address HAIs”* (Health professional; 20).

### Giving Authority to Infection Control Committees

Moreover, participants were of the view that committees responsible for controlling hospital infections (with support from hospital administrators and directors) should be granted the opportunity and authority to make independent decisions regarding the root causes of infections, and preventive mechanisms:

*“….Committees in charge of control of infections within hospitals should have the executive power to perform their duties. They should be in a position to question surgeons who refuse to adhere to medical guidelines, such as the hand hygiene guideline etc. Currently, the most important entities for HAI elimination are the infection control committees but they do not have any executive power to punish surgeons who violate the infection control guidelines”* (Health technical officer; 21).

### Increasing Financial and Material Resources

Almost all policy-makers emphasized the importance of funds – ie, adequate budgets for the control of infections. According to our participants, adequate allocation of health funds can serve as a key policy tool to facilitate programs seeking to develop human resources in this area. In addition, participants suggested that adequate financial resources could help in the provision and maintenance of equipment such as adequate masks, high-quality gloves, ventilation systems, anti-bacterial powder, disposable dishes and sheets, and laboratory equipment: *“Adequate funding can help establish provincial reference laboratories and provide equipment to enable them to curb the burden of HAIs”* (Health professional - head; 6). As a result, stakeholders recommended *a “separate budget to be allocated to control HAIs, on a yearly basis”* (Health professional - head; 18).

### Strengthening Information and Reporting System

Stakeholders also pointed out that good governance and effective prevention and control of HAIs is only possible when there is access to good information. Thus, a well-functioning national surveillance or reporting system, and monitoring and evaluation mechanisms, are critical components of the effective control of HAIs. Considering HAIs’ prevention and control in the accreditation of hospitals and quality improvement programs:

“*There should be a plan for follow-up after discharge – ie, a good monitoring system and information system to help detect and control HAIs, and to minimize under-reporting. At present, we do not know whether a patient who is discharged from hospital has picked up any infection or not, unless he/she is referred again*, *so planning for any intervention does not often happen*” (Policy-maker; 2).

*“Continuous monitoring and evaluation should be considered as core elements in the control of HAIs. Following up patients after discharge for at least ten days can help detect possible infections. This could enable us to come up with a more realistic rate of HAIs and solutions. However, assigning scores to accredited institutions, especially hospitals, can also be beneficial”* (Health professional; 11).

### 1-2. Enhancing Stewardship

Stewardship is defined in the World Health Report 2000 as “the careful and responsible management of the well-being of the population.”^15^ Enhancing stewardship would be very important for HAI prevention and control in Iran. Appointing a unique steward, improving interdisciplinary cooperation, influencing healthcare professionals’ behavior, and developing national guidelines and standards to control HAIs are some of the recommendations made for improving the stewardship of HAI prevention and control.

### Appointing a Unique Steward and Improving Interdisciplinary Cooperation

Participants added that structural reforms were subject to strong powerful forces and would therefore require good leadership and managerial support, especially from the MoH, but also from other organizations. According to these opinion leaders, one of the major ways to control HAIs is to appoint leaders who are solely responsible for steering everything related to HAIs, and coordinating the intersectoral activities of other departments, such as the Department of Health’s cooperation with the Department of Treatment, and the Environmental Health Department’s collaboration with the Infection Control Committee. In addition, interviewees mentioned the need for cooperation between surgeons, experts in HAIs and the infection control committee:

*“Control of HAIs is an intersectoral activity which demands a series of preventive and treatment strategies, requiring strong interdisciplinary and departmental collaboration within and beyond the MoH. The Food and Drug Administration, Research and Education Deputy, as well as the Deputy of Management and Resources do have important roles to play. These sectors, in turn, require stewards to coordinate all the activities within the country”* (Policy-maker; 1).

### Influence on Healthcare Professionals’ Behavior

Participants further indicated that the control of HAIs to a broader extent would require a positive change in attitudes, especially towards ensuring hand hygiene, access to equipment, public awareness, and an enabling work environment:

*“We need a positive change in attitudes. Healthcare providers should adhere to practices that will prevent the transmission of HAIs, for which they need an enabling environment. In a healthcare setting, when officials such as the chief, the administrator, the infection control supervisor and famous surgeons fail to show any compliance with infection control guidelines, instructions or protocols, we should not expect other personnel, let alone students, to obey those rules. Also, when patients are enlightened about HAIs, maybe they will tell doctors and nurses, for example to wash their hands before any contact or as they move from one patient to another”* (Technical officer; 22).

### Developing National Guidelines and Standards to Control Healthcare-Associated Infections

According to the participants, national guidelines also play a significant role by providing more accurate measures of controlling infection. Therefore, *“we (policy-makers) should provide the necessary instructions and guidelines to be adhered to by all hospital departments”* and systems to ensure that standards are met.

### 2. Strengthening Human Resources Policies

#### 2-1. Increasing Human Resources

Human resources are central to achieving successful prevention and control of HAIs but there is a limited workforce, especially nurses working in the public hospitals. According to our interviewees, when adequately matched to the number of beds, nurses can enhance the prevention of HAIs. Thus, the opinion of the stakeholders was that increasing the number of infectious disease specialists, microbiologists (in various laboratory diagnostic departments), and trained housekeeping staff, and offering them continuous training, could help reduce the spread of HAIs:

*“The Deputy of Management and Resources Development in the MoH should ensure that the number of nurses and other staff responsible for controlling infections is adequate for the number of beds. For example, some hospitals (whether with a 900-bed or 68-bed capacity) have only one infection control supervisor. The MoH should provide some facilities for applying the global standard for the quantity of healthcare personnel, and there is even a need to specify a local standard for the number of nurses per patient. We sometimes see a nurse expected to care for six patients, so how do we expect them to comply with infection control guidelines?”* (Health technical officer; 8).

### 2-2. Training/Education of Healthcare Personnel

Participants also pointed out the importance of bridging the knowledge gap through education and research. According to them, education (of various forms, eg, face-to-face provision of educational pamphlets and posters on infection control) and active engagement with the media can help improve people’s attitudes when it comes to adhering to infection control strategies such as hand hygiene, standards, safety precautions and the appropriate use of injections. Interviewees further suggested a “Hand Hygiene Day” to raise public awareness on the importance of hand hygiene, especially within hospitals:

*“….Control of infection should be included in the medical education curriculum and should be run by experts. Moreover, this should be done within the hospitals, too. University education should start this process and continuous education should follow”* (Health professional; 13).

### 2-3. Giving Rewards and Punishments

Additionally, participants indicated that Iran could make significant strides in controlling HAIs if healthcare providers were incentivized to adhere to standards or punished for failure to act in accordance with principles:

*“As there is a penalty for drivers who drive recklessly, in the same way there should be laws to punish those who do not adhere to guidelines and principles aimed at preventing HAIs, eg, hand washing. Penalties could be in the form of annual deductions from salary”* (Health professional; 15).

### 3. Appropriate Prescription and Usage of Antibiotics

#### 3-1. Antimicrobial Management Programs

Interviewees indicated that one of the major obstacles to the control of HAIs is antimicrobial resistance. However, according to our study participants, establishing antibacterial stewardship, a surveillance system and a comprehensive database would limit the needless prescription of antibiotics. The provision of evidence-based guidelines for antibiotics and the determination of patterns regarding their usage were also identified as essential:

*“Nowadays, irrational prescribing by specialists and even by general physicians, and also the use of antibiotics by patients, is very common in our country, so establishing antimicrobial stewardship and surveillance systems in the country will help curtail the excessive prescription and usage of antibiotics, thereby preventing antimicrobial resistance”* (Health professional; 10).

#### 3-2. Restriction of Antimicrobial Prescription

Participants also recommended that physicians should be made to consult infectious disease specialists before prescribing any form of antibiotics:

“*Physicians should not be allowed to prescribe all antibiotics, regardless of whether for prophylaxis or for treatment. Antibiotics should be categorized for prescribing. Furthermore, policy-makers and decision-makers should make a drug policy for compulsive consultations in hospitals. There is a need to involve pharmacologists and infectious disease specialists in these policies”* (Health professional; 18).

### 4. Environmental Sanitation and Personal Hygiene

#### 4-1. Environmental Sanitation

Furthermore, participants were of the view that proper sanitation practices (ie, separation of liquid from solid waste) and the use of disinfectants play a vital role in controlling HAIs. Besides this, measures to provide proper ventilation in specialized care units, such as within the operating room, should be considered:

*“Separation of hospital waste (solid from liquid) and proper disposal of waste, in addition to personal hygiene, daily cleaning and dusting, are important factors to be considered regarding control of HAIs”* (Health professional; 14).

### 4-2: Personal Hygiene

Participants expressed the view that the practice of personal hygiene (particularly hand hygiene) and compliance with standard precautions are very effective in the control of HAIs:

“*Personal hygiene, including hand hygiene, using gloves and masks, and all the standard precautions taken by all healthcare personnel, are very important for HAI prevention and control*” (Technical officer; 16).

## Discussion

This study presents four main themes explaining the measures that can be taken to control HAIs, as expressed by policy-makers: strengthening governance and stewardship; strengthening human resources policies; appropriate prescription and usage of antibiotics; and environmental sanitation and personal hygiene.

Identification of HAIs is a vital step needed in order for policy-makers to understand and design appropriate programs to address the problem,^6^ and may require reliable information or reporting systems.^16^ Hospitals with surveillance systems have shown low rates of HAIs.^3^ In Iran, although there is a national nosocomial infection surveillance program, significant gaps, especially with regards to the under-reporting of HAIs, do exist.^6,17^ In Mongolia, a national program for the establishment of a surveillance system for HAIs, with improved laboratory-based monitoring, was approved by the government in 2002. However, inadequate support from stakeholders and a shortage of resources and trained professionals delayed the policy implementation. The highest percentage of HAIs is estimated to be 0.05% in tertiary hospitals in the capital city of Mongolia, but underestimation has been declared very probable.^18^ In a study assessing the efficacy of surveillance of nosocomial infection at a general surgery, the incidence rate decreased from 18.4 to 14 per 1000 patient-days.^19^ Standards for accreditation can help institutions, especially hospitals, to meet the minimum safety requirements, and adhere to guidelines for the prevention of HAIs. In research conducted by Doshmangir et al regarding medical errors in Iran, the lack of a health information system is mentioned as one of the challenges in combatting medical errors, in line with the findings of this article.^20^

Stakeholders worldwide have also expressed the importance of interdisciplinary measures,^3^ good governance and effective managerial support in controlling HAIs.^21^ Based on the opinions of our study participants, Iran could achieve a significant reduction in HAIs if the issue of HAIs were prioritized on the national health agenda, as has been done in other countries, such as the United States where the Department of Health and Human Services (HHS) is responsible for HAI initiatives.^22^ A lot of studies have indicated that multiple interventions (such as hand hygiene, implementing surveillance systems, patient engagement, laboratory collaborations, environmental sanitation, antimicrobial stewardship, etc) are necessary for the prevention of HAIs,^3,23-25^ as well as government commitment and managerial support.^21^ A piece of research conducted in Lebanon indicated that, through interdisciplinary interventions, CAUTIs decreased by 83% in ICUs. ^26^

Adherence to guidelines can lead to a reduction in HAIs.^3,27^ Yet, despite its numerous benefits, adherence to public health guidelines is often low for several reasons, including a lack of awareness and incentives.^28^ An Antimicrobial Resistance Prevention and Control (ARPAC) study indicated that, in European hospitals, written guidelines promoted hand hygiene in 89% of hospitals.^29^

Inadequate physician and patient education regarding HAIs also have negative implications for their control.^30^ That is, people, when motivated, exhibit positive attitudes in their efforts towards achieving standards set to control HAIs.^16,31^ A typical example is incentivizing healthcare providers to wash their hands or use sterilants before attending to patients.^32^ In some studies, a lack of incentives is mentioned as a challenge to the prevention of HAIs, and asking managers to support the healthcare professionals is recommended as a solution.^33^ In some US states, such as New York, Pennsylvania, and South Carolina, there are policies aimed at motivating healthcare personnel, such as financial penalties, financial incentives, and financial support for the control of HAIs.^34^

Human resources play a central role in the achievement of improved health, including the prevention and control of HAIs. For example, an increase in the number of health staff and an improved nurse-to-patient ratio can reduce HAI acquisition rates and vice versa.^3^ In Iran, the workforce in public hospitals is limited, especially when it comes to nurses.^17^ Due to this, some hospitals (whether of a 900-bed or a 68-bed capacity) have only one infection control supervisor,^17^ instead of the recommended one nurse per 100 beds in acute wards and one per 150-250 beds in long-term care.^30,35^ As a result, almost all policy-makers emphasized the importance of funds – ie, adequate budgets for the control of infections. According to our participants, adequate allocation of health funds can serve as a key policy tool, facilitating programs that seek to develop human resources. This, they anticipate, could help increase the number of infectious disease specialists and microbiologists (in various laboratory diagnostic departments), and the provision of continuous training to reduce the spread of HAIs. Moreover, participants suggested that adequate financial resources could help with the provision and maintenance of equipment such as adequate masks, high-quality gloves, ventilation systems, anti-bacterial powder, disposable dishes and sheets, and laboratory equipment.

Patients and workers in healthcare settings often encounter a series of workplace hazards, which make them vulnerable to acquiring HAIs.^36,37^ Poor-quality water systems, a lack of good ventilation systems in specialized care units such as operating rooms, and inappropriate use of disinfectants, all threaten the safety of patients.^17,37,38^ Besides this, the availability of material resources (such as hand-rub dispensers) and facilities influence people’s attitude towards the prevention of HAIs.^3,39^ However, an increase in knowledge and awareness, through education,^40^ can lead to a positive change in attitudes and a reduced rate of HAIs,^3^ but with a bigger impact when the top management adheres to the guidelines.^41,42^ Appropriate use of antibiotics and the restriction of antibiotic prescribing are also key issues in tackling HAIs, so developing and implementing antibiotic stewardship programs is required for every health system.^43^

### Strengths and Weaknesses of Our Study

Our study faces certain limitations. Although we collected data from different levels and different places engaged in the prevention and control of HAIs (MoH, medical universities, and hospitals) to enhance the comprehensiveness and credibility of our findings, and we made efforts to use other methods such as focus group discussions, gathering together all the participants from the three levels was not feasible and in the end the views of the stakeholders were obtained through semi-structured interviews.

## Conclusion

In the view of the stakeholders, the elimination of HAIs demands interdisciplinary initiatives. According to our findings, success can be assured through good information and a follow-up system, enhanced governance and stewardship, adequate human resources and persistent training mechanisms, and appropriate prescription and usage of antibiotics. Moreover, there is a need to increase the awareness and knowledge of patients and healthcare workers regarding HAIs and their devastating consequences. While the ultimate recommendation of the key informants is that HAIs be placed among the priorities of the national agenda, financial commitment and the creation of an enabling work environment in which both patients and healthcare workers can practice personal hygiene, could also lead to a significant reduction in HAIs in Iran.

## Acknowledgements

Authors wish to thank the Deputy of Research and Technology of Tehran University of Medical Sciences, Tehran, Iran for financial support of this project (contract No. 93-02-27-25156). We also thank study participants for their contribution. We thank Mr. Abraham Assan for his scientific remarks.

## Ethical issues

The study was approved by the ethics committee of Tehran University of Medical Sciences, Tehran, Iran. Participation was voluntary. Besides this, we explained the research objectives, the method of data gathering, and the role of the researchers and then obtained informed consent from all study participants, and assured them of the confidentiality of any information they might provide.

## Competing interests

Authors declare that they have no competing interests.

## Authors’ contributions

Conception and design: AR, AE , AAS, and ARF; acquisition of data: AE and HS; analysis and interpretation of data and drafting of the manuscript: AE, AAS, and HS; critical revision of the manuscript for important intellectual content: AE, AAS, HS, HMA, AR, and ARF; statistical analysis: AE, AAS, and HS; obtaining funding: AE and AAS; administrative, technical, or material support: AE, AAS, HS, AR, HMA; supervision: AAS.

## Authors’ affiliations

^1^Bushehr University of Medical Sciences, Bushehr, Iran. ^2^Department of Health Management and Economics, School of Public Health, Tehran University of Medical Sciences, Tehran, Iran. ^3^Center for Communicable Diseases Control, Ministry of Health and Medical Education, Tehran, Iran. ^4^Research Center of Pediatric Infectious Diseases, Rasoul-e-Akram Hospital, Iran University of Medical Sciences, Tehran, Iran. ^5^Department of Epidemiology and Biostatistics, School of Public Health, Tehran University of Medical Sciences, Tehran, Iran.

## Key messages

**Implications for policy makers**

- To achieve a significant reduction in healthcare-associated infections (HAIs), their prevention and control should be at the heart of the national policy agenda.
- Prevention and control of HAIs in Iran require stronger governance, and the appropriate prescription and usage of antibiotics.
- Strengthening human resources policies and creating a favorable environment in healthcare settings can lead to an increase in adherence to standards and the control of HAIs.

**Implications for public**

Healthcare-associated infections (HAIs) are a major threat to public health. An increase in public awareness and the knowledge of patients and healthcare workers regarding HAIs and their devastating consequences, and the creation of an enabling work environment in which both patients and healthcare workers can practice personal hygiene can lead to a significant reduction in HAIs.
